# AKT/GSK3β Signaling in Glioblastoma

**DOI:** 10.1007/s11064-016-2044-4

**Published:** 2016-08-27

**Authors:** Ewelina Majewska, Monika Szeliga

**Affiliations:** Department of Neurotoxicology, Mossakowski Medical Research Centre, Polish Academy of Sciences, 5 Pawińskiego Str., 02-106 Warsaw, Poland

**Keywords:** Glioblastoma, AKT, GSK3β, Therapeutic target

## Abstract

Glioblastoma (GBM) is the most aggressive of primary brain tumors. Despite the progress in understanding the biology of the pathogenesis of glioma made during the past decade, the clinical outcome of patients with GBM remains still poor. Deregulation of many signaling pathways involved in growth, survival, migration and resistance to treatment has been implicated in pathogenesis of GBM. One of these pathways is phosphatidylinositol-3 kinases (PI3K)/protein kinase B (AKT)/rapamycin-sensitive mTOR-complex (mTOR) pathway, intensively studied and widely described so far. Much less attention has been paid to the role of glycogen synthase kinase 3 β (GSK3β), a target of AKT. In this review we focus on the function of AKT/GSK3β signaling in GBM.

## Glioblastoma

Glioblastoma (GBM), WHO grade IV, is the most common and aggressive of primary brain tumors. The prognosis for patients with GBM is poor, as the median survival time of patients with newly diagnosted GBM is 9.7 months [1]. The standard treatment of GBM relies on surgical resection followed by radiotherapy or combined radiotherapy and treatment with alkylating agents, mainly temozolomide (TMZ) [2]. Side effects of each treatment cause a significant decrease in quality of life and despite advances in standard therapy, less than 10 % of GBM patients are alive at 5 years [1]. Growing body of evidence suggests that glioma stem cells (GSCs), which possess the ability to self-renew and multilineage differentiation, play a significant role in angiogenesis, invasion, recurrence and resistance to chemo- and radiotherapy [3, 4]. Moreover, co-existence of different GSCs types in one GBM contributes to cellular heterogeneity, one of the causes of the failure of molecularly targeted therapies [3]. Thus, greater understanding of both GBM and GSCs biology may lead to the development of novel targeted therapies. Deregulation of many signaling pathways involved in growth, proliferation, survival, migration and apoptosis has been implicated in pathogenesis of GBM. One of these pathways is phosphatidylinositol-3 kinases (PI3K)/protein kinase B (AKT)/rapamycin-sensitive mTOR-complex (mTOR) pathway, intensively studied and widely described so far (for an exhausting review see [5, 6]). Less attention has been paid to the role of glycogen synthase kinase 3 β (GSK3β), a target of AKT.

## AKT Signaling in GBM

**Fig. 1:**
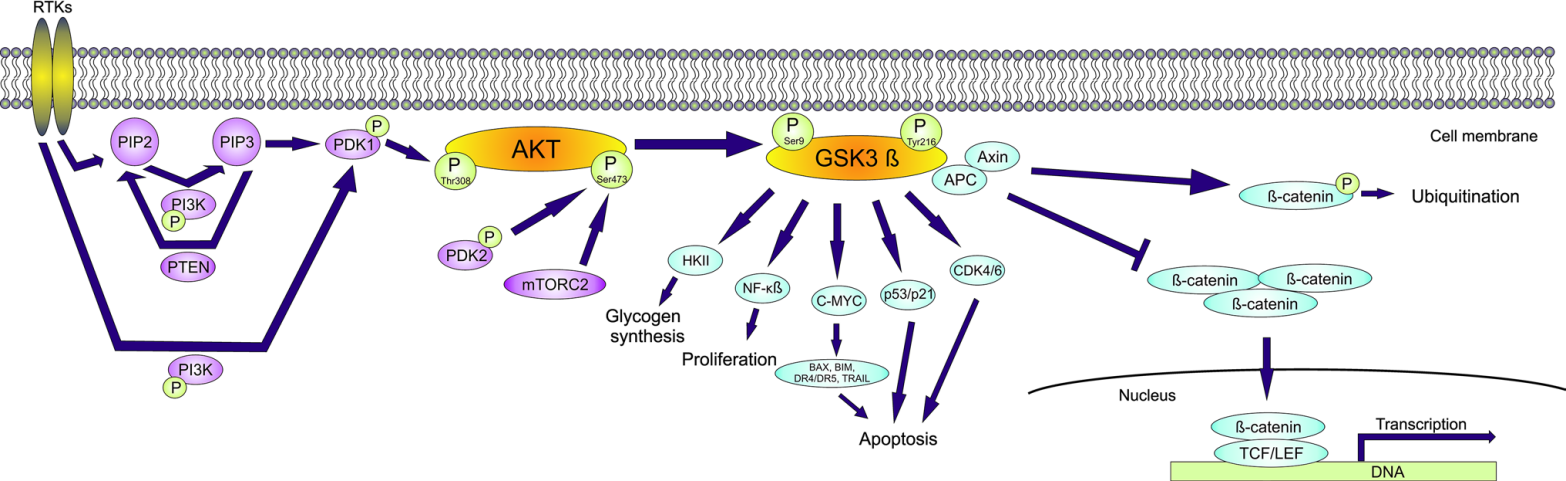
Interactions of the AKT signaling pathway with the GSK3β signaling pathways. The AKT signaling pathway is indicated in *purple*. The signaling pathways dependent on GSK3β are indicated in *blue*. High level of AKT phosphorylation triggers phosphorylation of GSK3β on Ser9 leading to its deactivation. Deactivation of GSK3β leads to translocation of accumulated β-catenin to the nucleus. By contrast, phosphorylation of GSK3β on Tyr216 causes its activation. Changes in GSK3β phosphorylation affect different downstream signaling pathways related to glycogen synthesis, proliferation, angiogenesis, apoptosis and transcription. (Color figure online)

AKT is a serine/threonine kinase activated by a dual regulatory mechanism that requires translocation to the plasma membrane and phosphorylation. AKT contains the pleckstrin homology (PH) domain that has a high affinity for the 3′-phosphorylated phosphoinositides 3,4,5-trisphosphate (PIP3). Phospholipid binding causes the translocation of AKT to the plasma membrane. PIP3 is generated by the addition of phosphate groups to phosphatidylinositol 4,5-bisphosphate (PIP2). This reaction is catalyzed by PI3K, thus PI3K activity is essential for the translocation of AKT to the plasma membrane [7]. PI3K can be activated by several mechanisms, all of which start with binding of a ligand to receptor tyrosine kinases (RTKs). Formation of PIP3 also results in translocation to the membrane and activation of phosphatidylinositol dependent kinases (PDK). PDK1 phosphorylates AKT on Thr308 what is both necessary and sufficient for AKT activation. However, maximal AKT activation requires additional phosphorylation at Ser473 by PDK2 or TORC2 complex of the mTOR [8–10]. The tumor suppressor phosphatase and tensin homolog (PTEN) inhibits AKT activation by dephosphorylation of PIP3 to PIP2 (Fig. 1) [11].

**Fig. 2:**
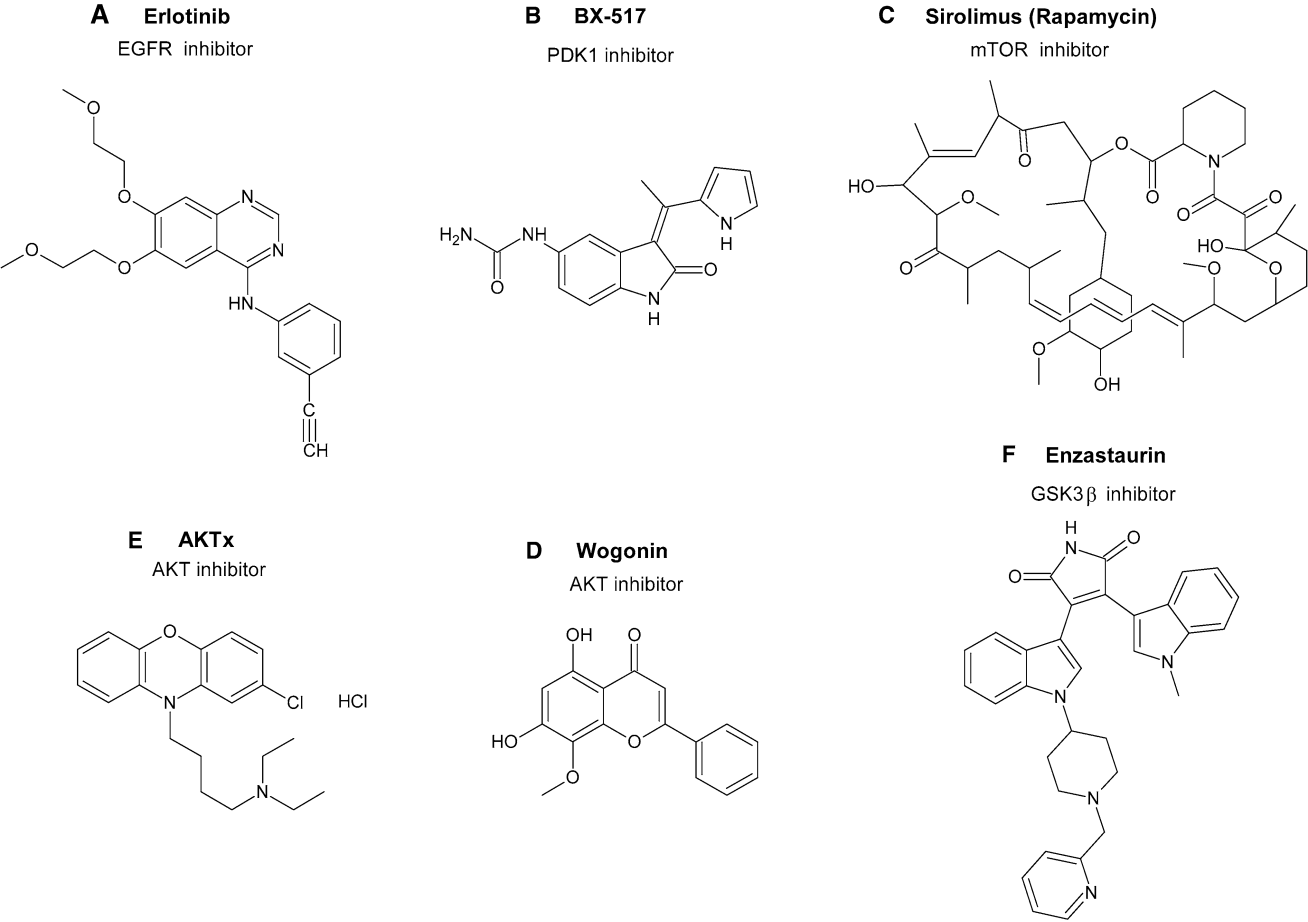
Structures of the selected inhibitors of the AKT/GSK3β signaling pathway

High level of phosphorylated AKT (p-AKT) has been reported to correlate with a poor prognosis for patients with GBM [12, 13]. A dominant mutation of genes coding for the AKT family members has not been identified in human tumor so far, therefore activation of AKT seems to be a consequence of the alterations of its upstream molecules [14]. Epidermal growth factor receptor (EGFR) belongs to RTKs and plays a crucial role in processes such as cell division, migration, adhesion, differentiation and apoptosis. *EGFR* amplification and/or overexpression occurs in 40–50 % of GBM [15, 16] and leads to the activation of PI3K/AKT signaling pathway in these tumors [5]. Activating mutations in *PIK3CA* and *PIK3R1* coding for subunits of PI3K have been identified in ∼10 % of GBM [17]. The other positive modulators of AKT activity, PDK1 and mTOR, are also upregulated in GBM, but evidence for mutations activating PDK1 and mTOR remains elusive. However, targeting of either of these molecules has emerged as a potential therapeutic strategy in GBM (Fig. 2a–c) [5, 17–20]. Upregulation of PI3K/AKT pathway has also been documented in GSCs. Preferential activation of this cascade relative to matched nonstem cells promotes the self-renewal and tumor formation of GSCs [21]. Thus, inhibition of PI3K/AKT/mTOR pathway has been proposed to be one of the strategies to target GSCs [22, 23].

The main negative regulator of AKT, PTEN, is often inactive in GBM due to gene mutation or methylation. Lack of active PTEN leads to an increased level of PIP3 and, in turn, an elevated activity of AKT [24, 25]. Latest findings indicate that a decrease in phosphorylation of AKT through PTEN may be obtain by suppression of miR-92b or miR-494-3p. Downregulation of these miRNAs increases expression of *PTEN* and decreases the level of phosphorylated AKT [26, 27]. Expression of both miR-92b and miR-494-3p is significantly increased in GBM tissues compared to normal brain tissues [27, 28]. Of note, loss of chromosome 10 resulting in the lack of *PTEN* has also been found in several GSCs lines [29].

## GSK3β Pathways in GBM

Once activated, AKT translocates to the various subcellular compartments where it phosphorylates several targets, including GSK3β, another multifunctional serine/threonine kinase. Ser9 is the phosphorylation site for AKT, and the phosphorylation of this residue leads to the inactivation of GSK3β. In contrast, phosphorylation of Tyr216 by autophosphorylation or by other tyrosine kinases increases the catalytic activity of GSK3β (Fig. 1) [30, 31]. The levels of GSK3β and GSK3β phosphorylated at Tyr216 were found to be increased in GBM as compared to the nonneoplastic brain tissues [32]. A growing body of evidence indicates that this protein is an important molecule influencing malignant phenotype of GBM. Initially, GSK3β was identified as a kinase that phosphorylated and inactivated glycogen synthase (GYS) [33], the final enzyme in biosynthesis of glycogen which is the main form of glucose storage [34]. Under basal conditions, GSK3β phosphorylates GYS suppressing its activity and blocking glycogen synthesis. Insulin stimulation activates the IR/IRSs/PI3K/AKT signaling cascade leading to the phosphorylation of GSK3β at Ser9. Inhibition of GSK3β results in activation of GYS and thereby glycogen synthesis [34]. The level of glycogen is particularly high in glioblastoma cell lines and accumulation of glycogen is phenomenon associated with growth of malignant cells [35, 36]. However, the role of GSK3β goes far beyond glycogen metabolism and glucose homeostasis. This protein plays a pivotal role in the modulation of activity of β-catenin, a coactivator of transcription factors belonging to the TCF/LEF (T-cell factor/lymphoid enhancing factor) family. β-catenin can be translocated to the nucleus where it binds to TCF/LEF proteins and activates genes encoding proteins involved in proliferation, differentiation, survival and apoptosis, such as: *MYC, MYCN, JUN, BIRC5* and *CCND1* [37–41]. Active GSK3β binds to axin and adenomatous polyposis coli (APC) proteins. This complex phosphorylates β-catenin, thus targeting it for degradation by the ubiquitination-proteasome system (Fig. 1) [42, 43]. In the absence of nuclear β-catenin, the TCF/LEF proteins recruit Groucho-related transcriptional repressors and block expression of target genes [44]. Both axin and APC are phosphorylated by GSK3β what increases the stability of the complex and the binding of β-catenin to it. Inhibition of activity of GSK3β promotes translocation of dephosphorylated and stabilized β-catenin to the nucleus [45].

GSK3β/β-catenin pathway is overactivated, and levels of c-Myc, N-Myc, c-jun, and cyclin D1 proteins are upregulated in GBM [41]. Besides the role in the modulation of β-catenin activity, GSK3β can also regulate stability and activity of nuclear factor-kappa B (NF-κB), an intracellular protein complex that controls DNA transcription and acts as a prosurvival factor [46]. Moreover, GSK3β phosphorylates c-MYC, a transcription factor implicated in the regulation of cell growth and proliferation [47]. Recent study suggests that GSK3β activity plays an important role in the regulation of GSCs survival and apoptosis [48].

## AKT and GSK3β as Therapeutic Targets in GBM

Upregulation of AKT/GSK3β pathways suggests that both AKT and GSK3β may be attractive therapeutic targets in GBM. Perifosine, an alkylphospholipid that inhibits AKT phosphorylation and activation reduced viability and proliferation of GBM cell lines by induction of autophagy [49]. In a mouse model of GBM this compound was not effective as a single agent, but it enhanced antitumor activity of CCI-779, an analog of rapamycin that inhibits mTOR [50]. The other inhibitors of AKT phosphorylation, AktX (Fig. 2d) and erufosine, also caused a significant growth inhibition of GBM cell lines or GBM xenograft tumors, respectively [51, 52]. Inhibition of AKT’s kinase activity by AktX resulted in a reduction of GSK3β phosphorylation which in turn activated GSK3β [51]. Several other studies have shown that AKT inhibitors indirectly influence the activity of GSK3β. Thus, inactivation of AKT by indomethacin-loaded lipid-core nanocapsules (IndOH-LNC) decreased phosphorylation of GSK3β activating this protein. Treatment of C6 and U138-MG GBM cells with IndOH-LNC induced apoptosis and arrested cells in G0/G1 phase [53]. Similarly, diminishing the level of phosphorylated AKT by wogonin (Fig. 2e) attenuated GSK3β phosphorylation at Ser9, downregulated β-catenin expression and suppressed proliferation of GBM cells [54]. In a very recent study, a 2-oxindole derivative was shown to inhibit PI3K/AKT pathway and its downstream effectors: CHK1, GSK3α, GSK3β and treatment with this compound reduced cell growth of GBM cells. Moreover, this compound decreased GSCs self-renewal and proliferation triggering both apoptosis and differentiation of the stem cell subpopulation [55].

Silencing of *GSK3β* or chemical inhibition of GSK3β activity induced apoptosis and reduced survival and proliferation of GBM cells in vitro and in vivo [45, 56, 57]. At the molecular level, GSK3β inhibition increased the level of tumor suppressors p53 and p21 in the cells carrying wild type *TP53* and was associated with downregulation of cyclin-dependent kinase 6 (CDK6) and decreased RB phosphorylation regardless of the cell genotype [32]. Of note, CDK6 is a component of the cyclin D-Cdk4/6 complex initiating RB phosphorylation which deactivates RB and leads to the progression of the cell cycle [58]. Downregulation of GSK3β with siRNA or with chemical inhibitors decreased an activity of NF-κB which in turn resulted in a decreased GBM cell survival in vitro and inhibition of tumor growth in vivo [45, 56]. Moreover, such manipulations resulted in c-MYC activation leading to the induction of expression of genes coding for apoptosis related genes: *BAX, BIM, DR4*/*DR5* and *TRAIL* [45]. Additionally, inhibition of GSK3β diminished the phosphorylation of GYS resulting in increased intracytoplasmic glycogen storage and decreased cytoplasmic glucose concentrations [45]. The influence of the direct inhibition of GSK3β on the phenotype of GSCs has recently been examined. TDZD-8, a non-ATP competitive inhibitor of GSK3β, inhibited GCS growth and capacity of self-renewal by the activation of the ERK/p90RSK pathway which led to the phosphorylation and inactivation of GSK3β [56].

The most promising compound reducing GSK3β activity is enzastaurin (LY317615), an inhibitor of protein kinase C-beta (PKC-β) (Fig. 2f). Enzasturin shows a direct inhibitory effect against GSK3β activity associated with the inhibition of GSK3β phosphorylation. This compound was clinically tested in a phase I and II trial in patients with recurrent GBM and it was well tolerated and presented antiglioma activity [59, 60]. Despite these encourage observations, phase III trials showed that enzastaurin is unlikely to be a useful agent in monotherapy because of its insufficient efficiency [61]. Therefore, the combination therapy of enzastaurin with radiotherapy, temozolomide and bevacizumab was investigated but showed no clear benefit for patients [62–65].

## Conclusions and Future Directions

In conclusion, the AKT/GSK3β signaling pathway plays a significant role in the pathogenesis of GBM. Moreover, mounting evidence suggests that it is implicated in GSCs survival. Thus, this cascade seems to be a promising target for creating new, more effective GBM therapy. Inhibitors designed to target various molecules belonging to AKT/GSK3β pathway seem to have enormous therapeutic potential. However, the modest efficacy presented by these compounds in the trials conducted so far suggests that they might be useful in the combination therapy rather than in the single-agent treatment. Clinical trials of combination of AKT/GSK3β pathway inhibitors with TMZ, radiotherapy and bevacizumab are ongoing.
